# Neuro-ophthalmological changes in healthy females exposed to a 5-day dry immersion: a pilot study

**DOI:** 10.1038/s41526-024-00344-0

**Published:** 2024-01-11

**Authors:** Marc Kermorgant, Thibault Chedmail, Fanny Varenne, Marie-Pierre Bareille, Arnaud Beck, Rebecca Billette de Villemeur, Pierre Fournié, Lisa Grondin, Ophélie Hélissen, Charlotte Membrives, Nathalie Nasr, Anne Pavy-Le Traon, Vincent Soler

**Affiliations:** 1 UMR INSERM U1297, Institute of Cardiovascular and Metabolic Diseases (I2MC), Toulouse, France; 2 Department of Ophthalmology, University Hospital of Toulouse, Toulouse, France; 3 Institute for Space Medicine and Physiology (MEDES), Toulouse, France; 4 Department of Neurology, University Hospital of Toulouse, Toulouse, France

**Keywords:** Physiology, Neuroscience

## Abstract

After exposure to microgravity, astronauts undergo microgravity-induced thoraco-cephalic fluid shift, which may lead to ocular changes called “spaceflight associated neuro-ocular syndrome” (SANS). The onset of SANS may be multifactorial, including a potential elevation in intracranial pressure. Moreover, little is known about the impact of spaceflight on SANS in women due to the fact that fewer female astronauts have spent time in long-term missions. The objective is to determine whether similar ophthalmological changes occur in healthy women after short-term exposure to microgravity. The auto-refractometer was used to determine objective refraction. The best corrected distance visual acuity was assessed with a Monoyer chart. The ocular axial length was assessed using optical biometry. The applanation tonometry was used to determine intraocular pressure. Peripapillary retinal nerve fibre layer thickness (pRNFLT), macular total retinal thickness, and ganglion cell complex (GCC) were measured using optical coherence tomography. Ocular axial length is reduced after DI. pRNFL is thickest after DI specifically in the temporal, temporal-inferior, and nasal-inferior quadrants. Macular total retinal at the inferior quadrant of the 6-mm ring is thickest after DI. Global GCC is thinnest after DI. In this study, 5 days of DI induces slight but significant ophthalmological changes in women. However, these subtle changes do not correspond to criteria defined in SANS.

## Introduction

The neuro-ophthalmological changes called Spaceflight Associated Neuro-ocular Syndrome (SANS) and their effects on the optic nerve are an important medical issue. After long duration flights, some astronauts present ophthalmologic modifications such as a globe flattening, an increase in retinal nerve fibre layer thickness (RNFLT) and total retinal thickness^1–5^. Studies have shown subclinical ophthalmological changes in some astronauts^5^, similar to idiopathic intracranial hypertension on Earth^6^. Studies show that several factors are involved, with possible localised events occurring at the level of the intra-orbital optic nerve with intracranial fluid accumulation^7–9^. However, even if exposure to microgravity does not pathologically elevate intracranial pressure (ICP), somehow it prevents the normal lowering of ICP when standing on Earth^10^. Moreover, little is known about the impact of spaceflight on SANS in women due to the fact that fewer female astronauts have spent time in long-term missions.

Analogues to microgravity such as dry immersion (DI) are essential models for determining the effects of spaceflight on astronauts’ bodies. DI mimics several aspects of spaceflight (support unloading, immobilisation, etc)^11^ and impacts a wide range of physiological mechanisms (alteration in cardiovascular system associated with sympathoexcitation^12^, possible impact on ICP^13–16^, etc). Few studies have determined the impact of DI on eye structure. However, our group observed an enlargement in optic nerve sheath diameter, an indirect surrogate marker of ICP, during short-term DI^15,16^. Ocular changes were also reported after head-down bed rest (HDBR)^17^, along with some isolated cases of optic disc oedema^3^. Although HDBR could be considered as an analogue for SANS^18^, DI may also emerge as a promising model for studying SANS. However, their impacts may differ in term of kinetics or mechanisms. Indeed, unlike HDBR, the head and the neck are still exposed to vascular hydrostatic pressure gradients due to gravity during DI^19^.

This study aimed to evaluate the neuro-ophthalmological modifications induced by a 5-day DI in women.

## Results

Eighteen healthy women had a mean of age: 29.2 ± 4.7 years, height: 164.8 ± 5.8 cm and weight: 59.3 ± 6.3 kg.

Compared to BDC (1.41 ± 0.21), distant visual acuity tended to reduce during R according to the Wilcoxon test (1.34 ± 0.19; *P* = 0.06). Compared to BDC (23.29 ± 0.97 mm), the Wilcoxon test revealed that ocular axial length was reduced during R (23.21 ± 0.92 mm; *P* < 0.01).

### Peripapillary retinal nerve fibre layer thickness

We observed a thickest global pRNFL (*P* = 0.03) during R, we also noticed a thickest RNFL both in the temporal (*P* = 0.04), temporal-inferior (*P* = 0.01) and nasal-inferior (*P* = 0.03) quadrants according to the paired *t* test (Table 1).

**Table 1:** Peripapillary retinal nerve fibre layer thickness mean for each quadrant before (BDC) and after (R) 5 days of dry immersion.

	BDC	R
pRNFLT (µm)	103.0 (97.3 to 108.7)	103.7 (98.0 to 109.4)*
TS (µm)	142.9 (131.7 to 154.1)	142.4 (131.3 to 153.4)
T (µm)	75.1 (69.7 to 80.6)	76.9 (71.3 to 82.5)*
TI (µm)	153.8 (142.5 to 165.1)	155.6 (144.7 to 166.5)*
NI (µm)	115.8 (105.3 to 126.2)	116.9 (106.3 to 127.4)*
N (µm)	77.3 (69.8 to 84.8)	77.5 (70.1 to 85.0)
NS (µm)	105.3 (95.9 to 114.6)	106.1 (96.5 to 115.6)

Data are means (95% CI).

*N* nasal, *NI* nasal-inferior, *NS* nasal-superior, *pRNFLT* peripapillary retinal nerve fibre layer thickness, *T* temporal, *TI* temporal-inferior, *TS* temporal-superior. *P < 0.05 vs. BDC.

### Macular total retinal thickness

The paired t test showed that macular total retinal thickness at the inferior quadrant of the 6-mm ring (mTRT-6-I) was thickest (*P* = 0.01) during R (Table 2).

**Table 2:** Macular total retinal thickness mean for each quadrant before (BDC) and after (R) 5 days of dry immersion.

	BDC	R
mTRT-1- (µm)	265.9 (254.9 to 277.0)	266.1 (254.8 to 277.4)
mTRT-3-S (µm)	344.7 (333.0 to 356.4)	345.2 (331.5 to 359.0)
mTRT-3-T (µm)	333.5 (327.4 to 339.5)	333.6 (327.7 to 339.5)
mTRT-3-I (µm)	344.9 (338.4 to 351.5)	345.8 (339.2 to 352.3)
mTRT-3-N (µm)	349.2 (342.0 to 356.4)	350.0 (342.9 to 357.2)
mTRT-6-S (µm)	312.5 (305.1 to 319.9)	312.9 (305.7 to 320.0)
mTRT-6-T (µm)	296.4 (289.8 to 303.0)	297.3 (290.7 to 303.8)
mTRT-6-I (µm)	297.6 (288.2 to 306.9)	298.7 (289.7 to 307.8)*
mTRT-6-N (µm)	325.5 (317.0 to 334.0)	325.9 (317.7 to 334.1)

Data are means (95% CI).

-1-, -3-, -6-, ring diameter (mm), *I* inferior, *N* nasal, *S* superior, *T* temporal, *TRT* total retinal thickness. *P < 0.05 vs. BDC.

### Ganglion cell complex

The quantification of GCC thickness was performed in 16 subjects due to technical glitches. We observed a thinnest global GCC (*P* = 0.02) during R, we also noticed a thinnest GCC both in the temporal-superior (*P* = 0.01) and temporal-inferior (*P* = 0.03) quadrants according to the paired t test (Table 3).

**Table 3:** Ganglion cell complex mean for each quadrant before (BDC) and after (R) 5 days of dry immersion.

	BDC	R
Global GCC (µm)	53.9 (51.9 to 56.0)	53.4 (51.4 to 55.5)*
S (µm)	54.8 (53.0 to 56.5)	54.8 (52.9 to 56.8)
TS (µm)	51.1 (48.8 to 53.4)	50.2 (47.8 to 52.5)*
TI (µm)	53.5 (51.4 to 55.7)	52.6 (50.1 to 55.1)*
I (µm)	54.6 (52.6 to 56.5)	54.2 (52.1 to 56.2)
NI (µm)	55.1 (52.7 to 57.5)	54.7 (52.4 to 56.9)
NS (µm)	54.7 (52.3 to 57.0)	54.5 (52.1 to 56.9)

Data are means (95% CI).

*GCC* ganglion cell complex, *I* inferior, *NI* nasal-inferior, *NS* nasal-superior, *S* superior, *TI* temporal-inferior, *TS* temporal-superior. *P < 0.05 vs. BDC.

### Intraocular pressure

Compared to BDC (14.1 ± 2.8 mmHg), IOP remained steady during R according to the paired t test (13.9 ± 3.3 mmHg; *P* = 0.80).

## Discussion

This study shows that 5 days of DI induces ophthalmological changes in women, such as 1) a diminished ocular axial length, 2) an increase in global pRNFLT as well in the temporal, temporal-inferior and nasal-inferior quadrants, 3) a rise in mTRT at the inferior quadrant, and 4) a thinnest global GCC as well in the temporal-superior and temporal-inferior quadrants. However, these slight ophthalmological changes did not reach thresholds like those observed in SANS.

Although the number of female astronauts spending time in long-term missions is still much fewer than their male counterparts, it would appear that they present modest visual alterations. Indeed, male astronauts have presented more severe ocular symptoms than female astronauts^1,2^. The protective effects of a higher vascular compliance and a slightly younger age in women have previously been put forward to explain these differences^20^. In either short- or long-duration spaceflight, degradation in distant and near visual acuity (hyperopic shift or residual choroidal folds) was observed respectively in 29 and 60% of astronauts^1,21^. A case report confirmed these findings. Indeed, a 57-year-old astronaut with 2 long-duration spaceflights, showed ophthalmological issues during the first mission (unilateral choroidal folds and a single cotton wool spot). These troubles had gotten worse during the second mission^22^. A rise in TRT in a healthy 45-year-old male astronaut was also documented after ~6 months on the International Space Station, and remained unresolved 1 year later^23^. A retrospective study using OCT scans has also revealed, in 15 astronauts, an increase in TRT compared to preflight scans. Postflight scans have also shown a greater global circumpapillary RNFLT ( ~ 2.9 µm) and the inferior quadrant particularly presented the greatest increase (~5.3 µm)^24^. Other studies showed similar trends. Indeed, in a longitudinal prospective cohort study during ~6 months on the International Space Station, 11 astronauts presented a persistent increase in global TRT over 1 year^4^. Interestingly, in a 1-year mission study, 2 of 11 crewmembers developed ocular changes with an increase in TRT. Choroidal folds and optic disc oedema persisted over 1 year^25^. Consistently with our previous study performed in similar conditions in DI in men^16^, we observed an increase in pRNFLT. In a previous study, spectralis OCT revealed an average increase in peripapillary retinal thickness (~5%) after a 30-day HDBR in a 25-year-old Caucasian male without noticeable presence of optic disc oedema^26^. However in similar conditions, another study described a rise in peripapillary TRT with cases of papilloedema^3^. Surprisingly, a 30-day HDBR induced major changes with a greater peripapillary TRT in healthy subjects than astronauts during similar duration spaceflight^27^. Moreover, the amount of optic disc swelling was dependent on HDBR duration where greater peripapillary retinal thickening, determined by OCT, was observed in a 70-day HDBR compared to a 14-day HDBR^17^. However in our study, the changes in OCT data were lower than those observed in HDBR and spaceflight studies. Time spent in DI condition probably explains these differences. Another assumption is that the semi-recumbent position would be less effective to increase ICP by limiting cephalad venous congestion in contrast to a strict supine position^27^. In our study, these subtle structural ophthalmological modifications might be due to a rise in ICP from microgravity-induced headward fluid shift.

Although there is a high interindividual variability, IOP seems to increase in the early phase of space mission and then returned to pre-flight values. Indeed, during an 8-day manned space mission, IOP determined by self-tonometry rose by 92% immediately after exposure to microgravity^28^. In a 10-day mission, IOP was dramatically increased by 114% in the early phase of the launch but returned to pre-flight values after the 3rd day^29^. A study gathering IOP data from 11 astronauts in 6 space shuttle missions confirmed this tendency. As suggested by Huang et al.^30^, the presence of cranial venous fluid shift does not prevent IOP to normalise over time, whether this be after short- or long-term exposure to microgravity. In our study, we did not observe any significant changes in IOP. These results are consistent with our previous study, where IOP was preserved in healthy male volunteers who underwent 5 days of DI^16^. In a 7-day HDBR, a decrease in IOP was observed during the 5^th^ and 7^th^ day in 8 female volunteers. This decrease in IOP would be correlated with cranial fluid shift-related hypovolemia induced by HDBR^31^. A case report also described a reduction in IOP in a 25-year-old Caucasian male after a 30-day HDBR^26^. Taibbi et al. compared ocular issues observed in 14- and 70-day HDBR and, although they described a slight increase in IOP in both cases, they did not find any differences in IOP levels during post HDBR^17^. Jóhannesson et al. hypothesized that if the balance between ICP and IOP is disturbed, this would favour optic nerve oedema^32^.

In all, DI induced slight ophthalmological changes in women, such as 1) a reduction in ocular axial length, 2) a thickest RNFL as well in the temporal, temporal-inferior and nasal-inferior quadrants, 3) a rise in mTRT at the inferior quadrant, and 4) a thinnest global GCC in the temporal-superior and temporal-inferior quadrants. These slight ophthalmological modifications were likely to be dwindled due to gravity always present above the neck. Thus, the cranial fluid shift may occur to a lesser extent in DI in contrast with a HDBR^19^. However, previous short-term DI studies performed in men showed an enlargement in ONSD reflecting indirectly a mild rise in ICP^15,16^. It is worth noting that ICP has been proposed to be the main cause of SANS and especially in the onset of optic disc oedema^5,33^. In our study, a 5-day DI was sufficient to induce small but significant ocular changes. Although these ocular changes did not reach thresholds like those observed during spaceflight, DI seems to be an encouraging terrestrial analog for studying SANS. It would also be interesting to assess ophthalmological changes in longer DI studies comparable to those carried out in HDBR (from 1 to 2 weeks).

## Methods

### Subjects

The clinical trial (ID-RCB 2021-A00705-36; Clinical Trial Identifier: NCT05043974) was conducted in accordance with the principles laid down by the Declaration of Helsinki after approval by both the CPP Ile de France II Ethics Committee (July 5, 2021) and the French Health Authority, ANSM (May 31, 2021). One subject could not complete the experiment due to technical issues and another subject was excluded for clinical trials regulatory reasons. Overall, 18 healthy women participated in the study and gave their written consent. The inclusion and non-inclusion criteria are presented in Table 4.

**Table 4:** Inclusion and non-inclusion criteria.

Inclusion criteria	Non-inclusion criteria
Healthy female participant, age between 20 and 40 years, height between 158 and 180 cm, body mass index (BMI) between 20 and 26 kg/m². Regular menstrual cycles and cycles lasting 20 and 35 days. Without oestroprogestative contraception (i.e., oral progestative contraception, intrauterine devices, implants or absence of contraception are allowed). No personal nor family record of a chronic or acute disease or physiological disturbances. Fitness level assessment: if age < 35 years: 35 ml/min/kg < VO_2_ max < 55 ml/min/kg. if age > 35 years: 30 ml/min/kg < VO_2_ max < 55 ml/min/kg. Active and free from any orthopaedic, musculoskeletal, and cardiovascular disorders. No history of regular smoking, no alcohol, no drug dependence, and no medical treatment (excepted of the aforementioned accepted means of contraception).	Past records of orthostatic intolerance, arterial hypertension, and cardiac rhythm disorders. Chronic back pains, vertebral fracture, scoliosis or herniated disc, history of knee problems, or joint surgery/broken leg. Past records of thrombophlebitis, family history of thrombosis, or positive response in the thrombosis screening procedure. Abnormal result for lower limbs in Doppler ultrasound. Bone mineral density: T-score ≤ −1.5, osteosynthesis material, presence of metallic implants. Poor tolerance to blood sampling and having donated blood (more than 8 ml/kg) in a period of 8 weeks or less before the start of the experiment.

### General protocol

**Fig. 1:**
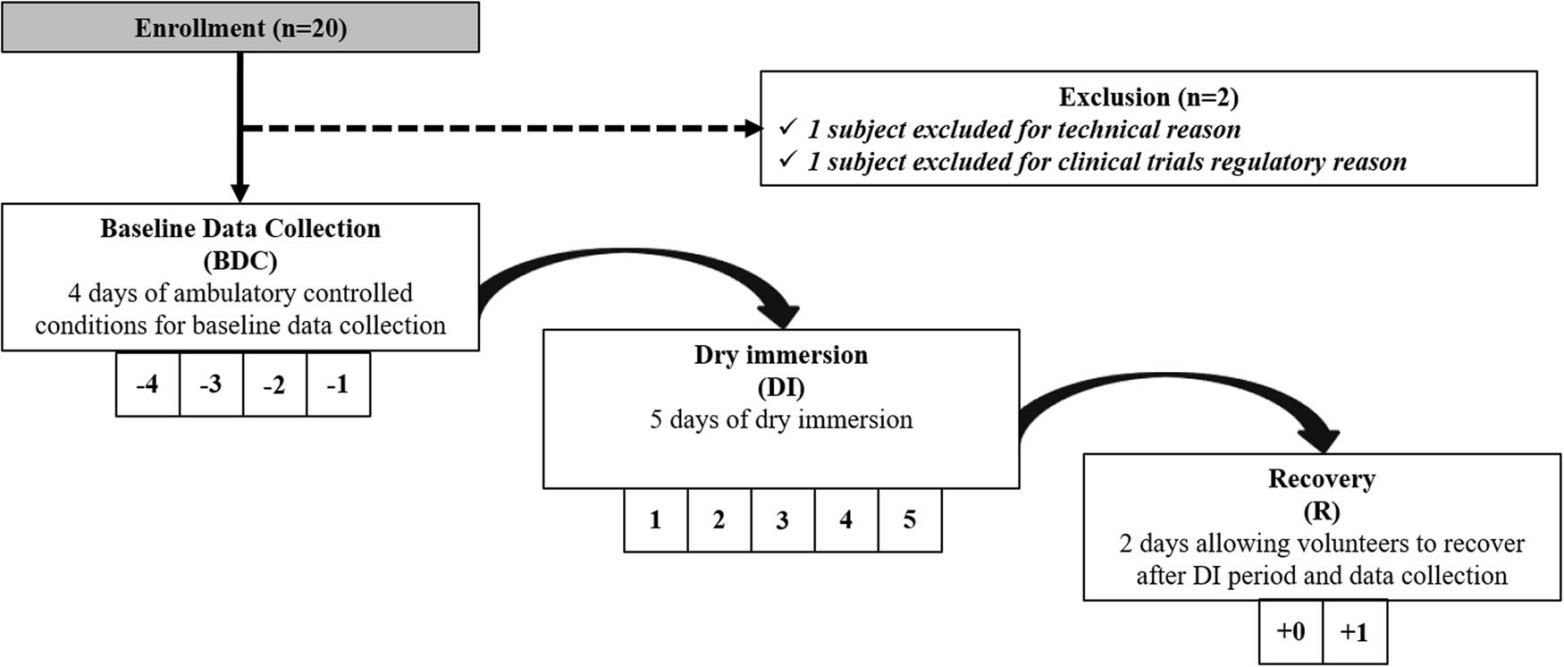
Flow chart of the study. The timeline of the 5-day DI experiment from the enrollment, baseline data colection (BDC), dry immersion (DI) period, to the recovery (R) phase.

From 20 September 2021 to 10 December 2021, 20 healthy females experienced 5 days of DI. This pilot study took place at the French Institute for Space Medicine and Physiology (MEDES). In summary, the protocol was as follows: 4 days of ambulatory control period before DI (BDC-4 to BDC-1), 5 days of DI (DI 1 to DI 5) and 2 days of ambulatory recovery (R 0 to R + 1). During the DI period, the subjects, first covered with an elastic waterproof fabric, were freely suspended in thermoneutral water (set from 32 °C to 34.5 °C) and remained in a semi-recumbent position. However, the subjects were allowed to get out of the bath in a 6° head-down position either for hygienic procedures or in supine position for specific measurements. Total out-of-bath supine time for the 120 h of immersion was 10.4 ± 1.5 h. Figure 1 represents a flow chart of the study.

### Ophthalmological measurements

**Fig. 2:**
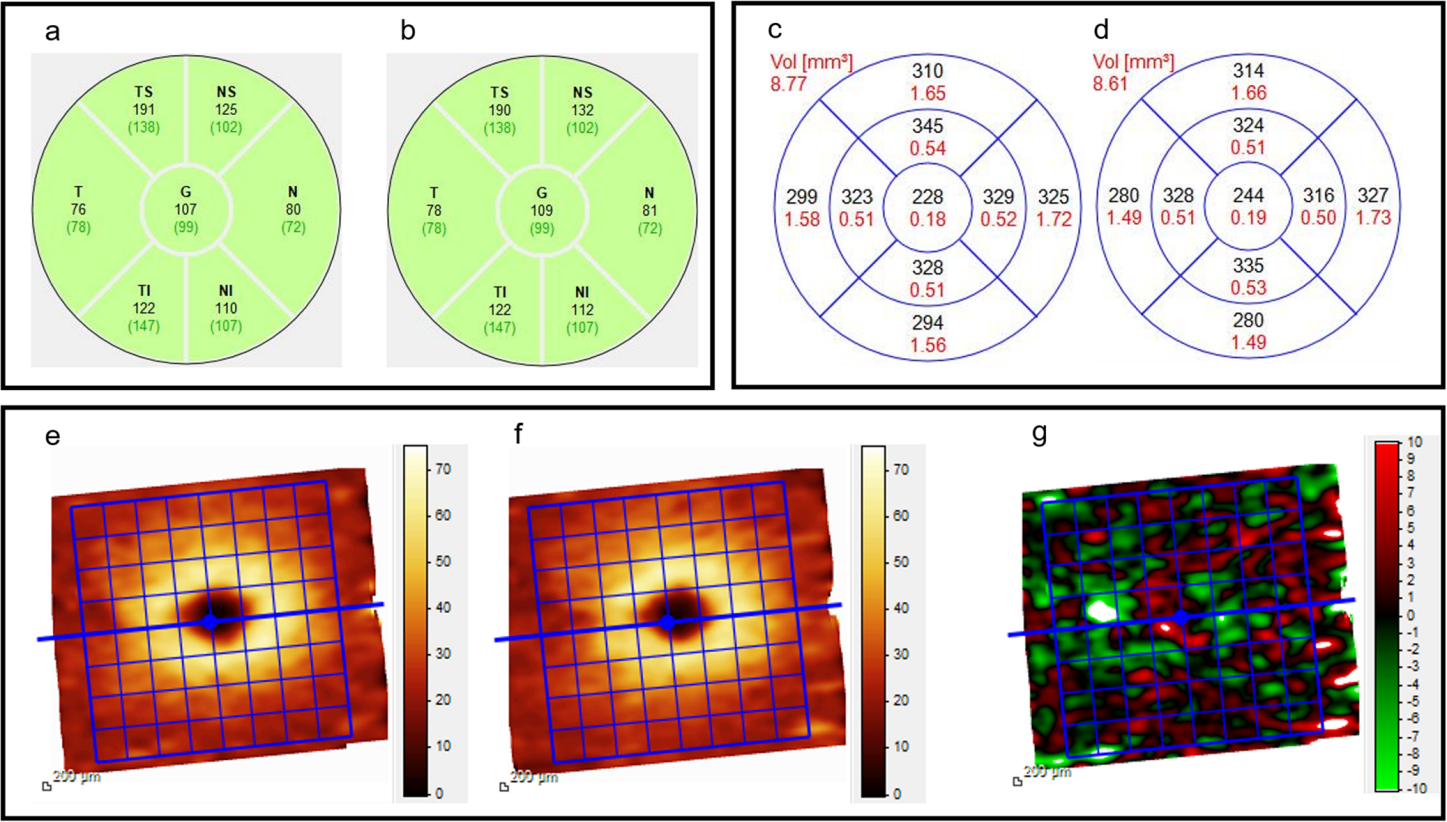
Spectralis OCT performed in the right eye from one subject. Peripapillary retinal nerve fibre layer thickness (black) and standard mean value relative to the age (green brackets) before (**a**) and after (**b**) DI. Macular total retinal thickness (black) and volume for each quadrant (red) before (**c**) and after (**d**) DI. Ganglion cell complex before (**e**) and after (**f**) DI with heatmap representing the thickness change (**g**). *G* global, *N* nasal, *NI* nasal-inferior, *NS* nasal-superior, *T* temporal, *TI* temporal-inferior, *TS* temporal-superior.

All subjects underwent a complete bilateral ophthalmologic examination before (BDC) and a few hours (5–6 h) after (R) DI, including objective refraction with the auto-refractometer (Tonoref III, Nidek SA, Gamagori, Aichi, Japan), assessment of best corrected distance visual acuity with a Monoyer chart, slit lamp biomicroscopy and dilated fundus evaluation. The examination also included: evaluation of the ocular axial length using optical biometry (IOL Master 500; Zeiss, Lena, Germany), applanation tonometry with a Goldmann® tonometer, pRNFLT and macular total retinal thickness (mTRT) measurements using optical coherence tomography (OCT) (Spectralis OCT, Heidelberg Engineering, Heidelberg, Germany). Retinal map and ganglion cell complex (GCC) images were acquired on a 6 × 6 mm mapping square centred on the fovea, with 5-µm resolution horizontal B-scans. RNFLT measures were acquired with circular-scan centered on the optic disc, and segmentation was verified manually. An example representing spectralis OCT data (pRNFLT, mTRT and GCC) before and after DI in 1 subject is described in Fig. 2.

### Statistical analysis

Ophthalmological data were expressed as mean ± SD or mean with CI 95%. The normality of the distributions was assessed with the Shapiro-Wilk normality test. Paired-*t* test and Wilcoxon tests (respectively for parametric and non-parametric data) were used to compare data. Differences were considered statistically significant when *P* ≤ 0.05. All statistical analyses were performed with GraphPad Prism 9 software.

### Reporting summary

Further information on research design is available in the Nature Research Reporting Summary linked to this article.

### Supplementary information

Reporting summary

## Data Availability

The data that support the findings of this study are available from the corresponding author upon reasonable request.
